# Does the Effect of Replacing Cottonseed Meal with Dried Distiller’s Grains on Nellore Bulls Finishing Phase Vary between Pasture and Feedlot?

**DOI:** 10.3390/ani11010085

**Published:** 2021-01-05

**Authors:** Alvair Hoffmann, Andressa Scholz Berça, Abmael da Silva Cardoso, Natalia Vilas Boas Fonseca, Maria Luísa Curvelo Silva, Rhaony Gonçalves Leite, Ana Cláudia Ruggieri, Ricardo Andrade Reis

**Affiliations:** Department of Animal Sciences, São Paulo State University, Jaboticabal, Sao Paulo 14884-900, Brazil; alvairtnn@hotmail.com (A.H.); abmael.cardoso@unesp.br (A.d.S.C.); nataliafonseca0531@gmail.com (N.V.B.F.); marialuisa1503@yahoo.com.br (M.L.C.S.); rhaonyleite@hotmail.com (R.G.L.); anaclaudiaruggieri@hotmail.com (A.C.R.); ricardo.reis@unesp.br (R.A.R.)

**Keywords:** by-product, finishing beef cattle, cottonseed meal

## Abstract

**Simple Summary:**

The use of less costly products that are not consumed by humans in animal feed has gained increasing attention in the context of sustainable production. Dried distiller’s grains (DDG), a co-product of the production of ethanol from corn, stands out for being efficient in the nutrition of ruminants, meeting both the energy and protein demands of the diets, when the cattle are kept in the pasture or feedlot. The study aimed to evaluate the effect of replacing cottonseed meal (CM) by DDG in two levels (50% (50DDG) and 100% (100DDG)), in terms of efficiency in the productive aspects of cattle finishing phase comparing pasture versus feedlot. The effect of replacing CM by DDG on dry matter, nutrients intake and nutrients digestibility depends on finishing system. While in the pasture system animal consumed more nutrients in the CM, a greater intake was observed in the 100DDG in feedlot. The nutrients digestibility was lower in the pasture. Animal performance and final body weight were higher in the feedlot. The use of DDG does not change the animal performance finished in pasture or feedlot, and it is a viable alternative to replace conventional supplements in finishing phase in both systems in tropical environment.

**Abstract:**

The study aimed to evaluate the effect of replacing cottonseed meal by dried distiller’s grains (DDG) in terms of efficiency in the productive aspects of beef cattle finishing in pasture versus feedlot. The experiment was conducted in a completely randomized design in a 2 × 3 factorial arrangement, with two production systems (pasture versus feedlot) and three supplements: CM, conventional supplement with cottonseed meal (CM) as a protein source; 50DDG: supplement with 50% replacement of CM by DDG; and 100DDG: 100% replacement. The effect of replacing CM by DDG on dry matter and nutrients intake and nutrients digestibility depends on the finishing system (*p* < 0.05). While in the pasture system animal consumed more nutrients in the CM, a greater intake was observed in the 100DDG in feedlot. The nutrients digestibility was lower in the pasture (*p* < 0.05). Animal performance and final body weight were higher in the feedlot (*p* < 0.0001), with averages of 1.57 kg/d and 566 kg of final body weight (FBW) for feedlot, and 0.99 kg/d and 504 kg FBW for pasture. The use of DDG does not change the animal performance finished in pasture or feedlot, and it is a viable alternative to replace conventional supplements in finishing phase in both systems in tropical environment.

## 1. Introduction

The finishing of cattle in feedlot or pasture is a strategy that allows intensive meat production through the exploitation of maximum biological efficiency, combined with the rapid deposition of muscle and fat tissue, which determine greater system productivity [1]. The finishing system, however, can be compromised by the inadequate nutrition of the animal in the growth phase, since the type and quality of the diet determine the supply of the animal’s requirements, which directly reflects on the performance and carcass quality [2]. Furthermore, the proportion of tissues in the carcass represents the most important aspect of animal composition, as it determines a large part of its economic value and influences the efficiency and cost of meat production [3]. Non edible byproducts from human feed are desirable to replace conventional sources of animal supplements to improve the sustainability of beef cattle production in tropical regions while maintaining productivity.

The diet quality also plays an essential role during the finishing phase in both systems, pasture or feedlot, as it determines the gains obtained by the animals. During the dry season, tropical grasses have low crude protein, higher fiber content and low forage allowance due to the seasonality of production, not providing the nutritional requirements to the animals [4]. In feedlot there is no such limitation, since feed is offered in quantity and quality according to the purpose of gain. In both systems, the productive response of animals occurs due to the intake, digestibility and metabolism of nutrients directly influenced by the type and quality of the ingredients used [5].

The use of less costly products that are not consumed by humans in animal feed has gained increasing attention in the context of sustainable production [6]. Cottonseed meal is a by-product of the oil industry and relatively rich sources of protein (30% to 50%) and amino acids [7]. Traditionally, it is used as a protein source in the ruminant nutrition as they tolerate well the presence of gossypol in the diet [8].

Alternatively, the dried distiller’s grains (DDG), a by-product of the ethanol production from corn or sorghum, stands out for being efficient in the nutrition of ruminants, meeting both the energy and protein demands of the diets, when the cattle are kept in the pasture or feedlot [9]. Although DDG are corn substitutes, the inclusion of this by-product is limited by seasonal availability and by negative impacts of excess N and sulfuric acid, which can affect animal performance, carcass quality and the environment [10].

In Brazil, most of the industries produce DDG without solubles, resulting in the dry grind processing of corn for ethanol production. The use of DDG can improve ruminal health due to its highly fermentable fiber, and low starch content, reducing the acidosis risk occurrence of cattle finished in feedlot with high proportion of grains [11]. DDG, moreover, consists of a source of minerals and has a high value of non-degradable protein, increasing the metabolizable protein supply [12]. However, although DDG have been used in temperate regions in *Bos taurus* animals’ diet, mainly in feedlots [10], there are few studies providing information about use of this by-product in tropical regions and comparing finishing systems in feedlot or pasture.

The study aimed to evaluate the efficiency of replacing cottonseed meal by DDG on intake, digestibility, daily weight gain, carcass gain and yield of Nellore cattle finished in pasture or feedlot. We hypothesized that DDG can replace cottonseed meal without compromises the productivity in both finishing systems (pasture or feedlot).

## 2. Materials and Methods

### 2.1. Location and Experimental Area

The study was conducted during the animal finishing phase, at the Forage and Grasslands sector of the Sao Paulo State University “Julio de Mesquita Filho” (FCAV/UNESP), Jaboticabal, SP, located at 21º15′22′′ South, 48º18′58″ West, at 595 m of altitude, climate is subtropical of the AW type according to the Köppen classification, characterized by warmer and rainfall summer, and dry winters.

Climate data was daily determined by the Department of Exact Sciences of FCAV/UNESP. During the experiment period, from April to August/2016, average of temperature was 20.6 °C, with a minimum of 12.6 °C and a maximum of 31.0 °C, and the average precipitation was 93 mm, totalizing 28 rainy days.

This experiment was conducted during the dry season, in which animals that came from the post weaning phase in pasture were finished in two different systems: pasture or conventional feedlot.

The finishing phase experiment was conducted after the post weaning phase in order to evaluate the nutritional history of Nellore bulls raised in pastures of *Urochloa brizantha* (Hochst ex A. Rich) Stapf cv Marandu (Marandu grass). During the post weaning phase, pastures of Marandu grass were managed at 25 cm height in continuous grazing and variable stocking rate, using put and take stocking technique [13].

Animals from the pasture finishing system were kept in the original paddock to minimize possible environment and stress effects and were adapted to finishing diet for 18 days, increasing 0.3% to 0.3% BW of the supplement amount until established its intake. After adaptation period, supplement was offered ad libitum once a day and a leftover of 3% to 5% was allowed.

At the beginning of the experiment, all animals were submitted to the control of endo and ectoparasites, using albendazole sulfoxide as vermifuge and fluazuron pour-on for cattle tick control. The experimental period was 117 days, from May to July 2016, with the first 18 days of adaptation to the diets, and three evaluation periods of 33 days each, for the pasture and feedlot systems.

All animals were submitted to three treatments in a completely randomized design in a 2 × 3 factorial arrangement, with two production systems (pasture versus feedlot) and three supplements: CM, conventional supplement with corn as an energy source and cottonseed meal (CM) as a protein source; 50DDG, supplement with replacement of 50% of the CM protein source by DDG; and 100DDG, supplement with 100% replacement of the CM protein source by DDG. The inclusion of corn DGG was determined on crude protein basis and its chemical composition is presented in Table 1.

### 2.2. Feedlot System

In the feedlot system, the effect of replacing cottonseed meals was studied using 3 pens per treatment, which was considered the experimental unit. Animals were allocated in collective pens of 60 m^2^, with concrete floor, and covered feeder area, totalizing 4 animals/pen. We used 36 Nellore bulls (*Bos indicus*), with an average of initial body weight (BW) of 409 ± 40 kg, distributed in three treatments (Table 2), receiving a diet with roughage:concentrate (R:C) ratio of 30:70 respectively, with corn silage being the source of roughage.

The diets were formulated for a gain of 1.5 kg/d, according to NRC [14]. The adaptation of the animals in the feedlot followed a stair-step protocol with variation in the R:C ratio, being five days with 65:35, five days with 50:50, five days with 40:60, and three days with 30:70, totaling 18 days of adaptation. Final diet was 30:70 of R:C and was offered ad libitum at 6 am and 3 pm. In the morning, leftovers were removed and weighted in order to adjust the quantity to allow daily leftovers of 3% to 5%.

In the feedlot system, feed intake was measured daily in each pen composed of 4 bulls by determining the difference between what was supplied and what was left over. In each experimental period, samples of leftovers were collected during 5 days, and later a composite sample was made for chemical analysis.

### 2.3. Pasture System

In the pasture system, the effect of replacing cottonseed meals was assed using 3 paddocks per treatment. The experimental area for evaluation of grazing animals was formed with *Urochloa brizantha* cv. Marandu (palisade grass), divided in 9 experimental paddocks, being three of 0.7 ha (3 animals) and six of 1.3 ha (4 animals). The stocking rate was kept the same (2.5 animal unit/ha; animal unit = 450 kg of BW) in all paddocks using additional animals, according to put and take stocking technique [13], in a continuous stocking system.

In this system, 33 Nellore bulls were used, with average of initial BW of 407 ± 33 kg and 24 months of age. Animals were distributed in three treatments (Table 3), receiving forage ad libitum and supplementation of 1.5% BW at 8 am, in open trough, with at least 0.5 linear meters per animal.

Animals adaptation to the supplement followed a stair-step protocol, changing the amount of concentrate, which the first five days was 0.7% BW, five days with 1% BW, five days with 1.3% BW and three days with 1.5% BW, totaling 18 days of adaptation.

### 2.4. Forage Samples, Leftovers and Roughage from Pasture Finishing System

Forage mass was measured every 28 days; 80 points of height of palisade grass were taken randomly in the paddock with the aid of a graduated ruler [15]. From the average height, three representative samples of grass per paddock were collected, by cutting 5 cm from the soil of all forage contained in a 0.25 m^2^ metallic frame [15].

The samples were initially weighed, then sub-sampled in 2 parts, one for determining the pasture morphological composition, manually separated into senescent material (leaf and stem), green stem (leaf sheath and stem) and green leaf blades, and one for green leaves to estimate of total dry matter (TDM, kg MS/ha) availability of forage from each paddock. The samples were dried in an oven with air circulation at 55 °C for 72 h and weighed.

Weekly composed samples of leftovers and roughage at the end of feedlot were taken to a forced ventilation oven at 55 °C for 72 h and then ground in a Wiley mill, with a 2 mm sieve, for sample removal for incubation and determination of indigestible neutral detergent fiber (iNDF) [16], and then ground to 1 mm for chemical composition analyses. The concentrate ingredients were sampled once in each experimental period, being stored in a freezer (−20 °C) for further laboratory analysis.

### 2.5. Analysis of Feed Samples, Leftovers and Feces

The feed, leftover and feces samples were quantified in terms of dry matter (DM, method 934.01), organic matter (OM, method 942.05), and ether extract (EE, method 954.02), according to [16]. Crude protein (CP) was obtained by thermal conductivity using the Leco^®^ equipment (model FP-528, Leco Corporation, St. Joseph, MI, USA). The neutral detergent fiber (NDF) and acid detergent fiber (ADF) evaluations were performed on the Ankom^®^ 2000 (Ankom Technologies, Macedon, NY, USA). The NDF of the concentrates, treated with thermostable amylase, the NDF, ADF, NDF corrected for ash and protein (NDFap), lignin (H_2_SO_4_ 72%), neutral detergent insoluble nitrogen (NDIN) and acid detergent insoluble nitrogen (ADIN) of forage were analyzed according to AOAC methodologies [17].

Non-fibrous carbohydrates (NFC) were determined according to [18].

### 2.6. Nutrients Intake and Digestibility in Pasture System

To estimate fecal production, we used chromium oxide (Cr_2_O_3_) as an external marker, the usual inert marker in research in Brazil and approved by local and national Ethics Committee on the Use of Animals. For this assay, 10 g/animal/d was provided at 9 am for 10 days via the esophagus with the aid of an applicator. The first 7 days were used for adaptation and the last 3 for collection of feces [19], which were collected at 7 am and 1 pm; 9 am and 3 pm; and 11 am and 5 pm, respectively, immediately after spontaneous defecation. For this evaluation, eighteen animals were used (BW = 500 kg), with 6/treatment and 2/paddock.

Fecal recovery of Cr_2_O_3_ was determined following the methodology of atomic absorption spectrophotometry [20]. From these data, fecal excretion (FE) was determined through the equation below [21] (Equation (1)):

(1) $$E\;\left(g/d\right)=\frac{Cr_{2}O_{3}\;\mathrm{provided}\;\left(g/d\right)}{Cr_{2}O_{3}\;concentration\;\left(g/kg\;DM\right)}$$

Concomitantly to the feces collection, simulated grazing collections were performed by the hand plucking method [22] to evaluate the chemical composition of the forage consumed by the animals.

Forage intake was estimated based on fecal production data and iNDF as an internal marker. A sample composed of feces was made based on the dry weight in air, per animal, of the three days of collection, identified and subsequently analyzed for chromium contents, by atomic absorption spectrophotometry [23], and quantity of nutrients as previously described.

The individual intake of the supplements was estimated according to the average supply of supplements for each animal in the paddock (1.5% BW).

From the intake of nutrients by forage and supplements and their excretion in feces, the total apparent digestibility was calculated through the calculation: DDM = (TDMI − FE)/TDMI where, DDM = total apparent digestibility of dry matter (%); TDMI = total dry matter intake (kg/d); FE = fecal excretion (kg/d).

The intake of total dry matter (TDMI), organic matter (OMI), crude protein (CPI), neutral detergent fiber (NDFI), indigestible neutral detergent fiber (iNDFI), non-fibrous carbohydrates (NFCI) and ether extract (EEI) were determined. Likewise, the digestibility values of total dry matter (TDMD), organic matter (OMD), crude protein (CPD), neutral detergent fiber (NDFD), non-fibrous carbohydrates (NFCD) and ether extract (EED) were determined.

### 2.7. Nutrients Intake and Digestibility in Feedlot System

The intake of animals from the feedlot was measured daily by the difference between the supply and the leftovers of each pen. The diet and orts of each pen were sampled weekly, making a composite sample at the end of the experimental period. The samples were dried in an oven at 55 °C for 72 h and then ground in a Wiley mill (Wiley Mill, Thomas Scientific, Swedesboro, NJ, USA), with 2 and 1 mm sieves.

At the end of each experimental period, feces were collected from the animals immediately after defecation to determine digestibility. The samples were collected at alternate times, 4 pm and 11 am, 3 pm and 09 am and 2 pm and 7 am on the first, second and third days of collection, respectively.

A sample composed of feces based on dry weight in air was constituted, through samples collected over the days and times of collection, identified and subsequently analyzed for the contents of iNDF [15], to determine the fecal excretion through an internal marker [24], and other bromatological analyzes.

### 2.8. Animal Performance

In order to determine the average daily gain (ADG) of the animals, weighing was performed at the beginning (initial body weight (IBW)) and end (final body weight (FBW)) of the experimental period, after a 12-h feed and water fasting. The same procedure was taken at the beginning and end of the adaptation period, in order to estimate its ADG (ADGadap). Intermediate weighing every 33 days, without fasting, was also conducted to adjust the supplement supply.

All animals were slaughtered in a commercial slaughterhouse. The carcass of each animal was divided into two half-carcasses, which were weighed to obtain the hot carcass weight (HCW). After weighing, carcass yield (CY) was determined as a function of fasting live weight and HCW.

The daily carcass gain (ADGc) was estimated according to the following equation (Equation (2)), considering the initial carcass weight (ICW) as 50% of the IBW fasting for 12 h.

(2) $$ADGc=\;\frac{HCW-ICW}{nº\;of\;days\;in\;finishing\;phase}$$

### 2.9. Statistical Analysis 

Animals were distributed in a completely randomized design, in a 2 × 3 factorial arrangement, with two production systems (pasture versus feedlot) and three supplements (CM, 50DDG, 100DDG). Supplements and the finishing system were considered fixed effects and pens and paddocks were considered random effects. The paddock was considered the experimental unit for finishing in pasture, and the pen for feedlot. Data analysis was performed using the statistical package SAS (2008), version 9.2, using mixed models by PROC MIXED. The averages generated were compared with the Tukey test using the PDIFF option in the LSMEANS command, when significant. The level of significance used to assess the differences between the means was α = 0.05.

## 3. Results

The results presented in Table 4, Table 5, Table 6 and Table 7 come from Dr Alvair Hoffmann’s PhD thesis [25].

The finishing system affected the intake of TDMI, CPI, NDFI and iNDFI (*p* < 0.05), which averages were higher in the pasture finishing system compared to feedlot. The OMI, NFCI and EEI were higher in the feedlot compared to pasture system (*p* < 0.05) (Table 4).

The TDMI in % BW was higher in animals supplemented with 50DDG (*p* < 0.05). The intake of DM, OM, CP, NDF, iNDF and EE were similar among feedlot diets (*p* > 0.05). In the pasture finishing system, the animals had lower TDMI in the CM (10.04 kg/d) in detriment to the 50DDG (12.08 kg/d). The OMI in the pasture system was lower in the CM (8.15 kg/d) compared to 50DDG and 100DDG, with averages of 10.24 and 9.54 kg/d, respectively. The CPI in the pasture system was lower in the CM (1.5 kg/d) compared to 1.74 and 1.66 kg/d of the 50DDG and 100DDG, respectively. The NDFI in the pasture system was lower in the CM (3.34 kg/d) compared to the 50DDG and 100DDG, whose averages were 4.92 and 4.43 kg/d, respectively. The iNDFI in the pasture system was higher in 50DDG (1.94 kg/d) compared to the CM and 100DDG, which averaged 1.36 and 1.44 kg/d, respectively. Metabolizable energy intake was higher in pasture compared to feedlot (*p* = 0.0409), besides being higher in the 50DDG diet than the other treatments (*p* = 0.0252) (Table 4).

Nutrient digestibility was influenced by finishing systems (Table 5). TDMD, OMD, CPD, NFCD and EED were higher in feedlot when compared to pasture finishing (*p* < 0.05). On the other hand, NDFD was lower in feedlot than in pasture, whose averages were 468.5 and 523.1 g/kg, respectively (*p* < 0.05). There was no difference among diets in both finishing systems for nutrient digestibility (*p* > 0.05) (Table 5).

Animal performance was higher in the feedlot finishing system (*p* < 0.0001) compared to pasture, with averages of 1.57 kg/d and 0.99 kg/d, respectively (Table 6). The FBW of animals finished in feedlot was higher (*p* < 0.0001) than animals finished in pasture, with averages of 566 kg and 504 kg, respectively. ADGc was also higher in feedlot (*p* < 0.0001) when compared to the pasture system, whose averages were 1.09 kg/d and 0.78 kg/d, respectively. Among diets, there was no difference for ADG (*p* = 0.3698) and ADGc (*p* = 0.2665). The ADG during the adaptation (ADGadap) was not influenced by finishing systems (*p* = 0.1571) and diets (*p* = 0.0661) (Table 6).

In the pasture finishing system, there were no differences in forage mass, forage allowance and morphological composition among treatments (*p* > 0.05), which averaged 6.0 t DM/ha of FM; 3.2 kg DM/ha of forage allowance; 8.0% of green leaf; 13.3% of green stem; 26.9% of senescent leaf and 26.3% of senescent stem, during dry season experiment (Table 7).

## 4. Discussion

### 4.1. Dry Matter and Nutrients Intake

The nutrients intake is one of the primary factors in the feed conversion into animal product, so that the intake of digestible DM is more affected by the DMI than by the digestibility itself [26]. In this study, although animals finished in pasture had higher TDMI, the animal performance was lower for this finishing system when compared to feedlot.

However, the higher TDMI of animals finished in pasture with a diet containing 50% DDG compared to animals receiving CM and the other animals finished in feedlot in both diets maybe could provide a higher rate of passage. As explained by the NRC [27], the digesta rate of passage through the digestive tract is directly related to feed intake, where greater intake promotes an increase in the passage rate of, influencing the digestibility of the ingested diet.

Differences in intake between finishing systems may be associated to the energy content of the diet, which reduces voluntary intake, as it promotes physiological satiety due to the animals’ energy demand, when diets contain high concentrated content with greater digestibility [5]. Diets with high concentrate content (above 75% in DM), low fiber content (below 25%) and high digestibility (above 66%) can result in lower TDMI, since energy requirements are met at lower levels of intake [5], a fact observed in animals finished in feedlot, which concentrate had approximately 132.6 MJ/kg DM of metabolizable energy (ME), while in pasture the supplement showed 145.4 MJ/kg DM of ME.

Additionally, animals under grazing conditions require 8.5% more metabolizable energy for maintenance than animals in feedlot, due to the higher harvesting and grazing energy costs [28]. In our study, the intake of ME was about 15% higher in pasture finishing system (1606.7 MJ/d) compared to feedlot conditions (1393.6 MJ/d), which is probably a consequence of the fiber content of forage. In both finishing systems, diets with CM or with 100DDG had the same TDMI. This result can show that replacing 100% of cottonseed meal by DDG does not affect or limit the intake of animals, even with the higher NDFI and EEI promoted by DDG, as reported by [29], due to its fat content, that vary from 8.8 to 12% DM [30].

### 4.2. Apparent Digestibility of Dry Matter and Nutrients

In both finishing systems, diets with CM or with DDG showed the same apparent digestibility of nutrients, except for NDFD, which was greater in 100DGG compared to CM and 50DDG. This fact may be explained by the supply of DDG in high intake supplements, used for finishing animals in pasture or feedlot, that can promote differences in the pattern of ruminal fermentation. The use of feeds with a high proportion of digestible fiber can bring benefits in the microbial protein synthesis and not negatively affect the use of fiber, due to the low lignin content [31].

The degradation processes and ruminal motility of the fiber are integrated events, since as the speed of use of the fibrous fraction increases, the necessary time for the particles to reach their specific point of removal is reduced [31]. This process is closely linked to the degradation rate of the potentially digestible fraction, because as its disappearance increases, the concentration of components of higher density represented by the indigestible fraction increases, accelerating its exit from the rumen [30], with potential increase in digestibility, as previously described for animals that received a diet with 100% DDG in the finishing phase.

The increase in digestibility may be related to a higher proportion of rapidly digesting tissues [32], higher protein levels and lower NDF content, in addition to the intake level, that is inversely proportional to digestibility, given that the increased TDMI reduces the rumen retention time of the feed and digestibility [33]. As they presented higher TDMI, animals receiving a 50% DDG diet did not have an affected digestibility compared to animals that received CM and this is due to the composition of DDG, mainly the fibrous fraction and its digestibility.

Although there is evidence of animal adaptation to the increase of indigestible components content in the diet through the expansion of the volume and pool of digesta resident in the rumen, limits are defined, beyond which the intake is reduced permanently [34]. Thus, the higher concentration of iNDF observed in the diet with CM (Table 3) may has been effectively related to the lower TDMI observed in animals receiving CM at the end, as it causes a greater effect of ruminal repletion. Possibly, the lower intake observed for these animals, in relation to animals receiving 100% DDG, is attributed to this principle, given its high iNDF content compared to other treatments (Table 3).

### 4.3. Animal Performance

Diets containing DDG present a different profile of the protein ingested, which has a high rumen undegradable protein (RUP) content, allowing to increase the amount of essential amino acids in the metabolizable amino acid pool [35], which may affect animal performance. However, the replacement of CM by DDG did not affect the productive responses, showing that this by-product can be used as a cheaper protein source without changing the animal performance.

Difference in feed planning between feedlot and pastures can alter the body composition of cattle, since the use of higher proportions of concentrate results in less participation of rumen content in relation to BW, increasing the carcass yield, and the opposite can be observed in diets with high participation of fibrous tissues [36]. This may explain the difference in body composition and changes in ADG, final BW and carcass weight between the finishing systems. Thus, the influence of the ruminal size in relation to the BW is emphasized, since the animals finished in pasture presented higher TDMI and higher NDFI.

The ADG during the adaptation (ADGadap) was not influenced by finishing systems and, as observed by [37], the animals did not show oscillation in intake during this period. However, the adaptation period of animals consuming diets with a high concentrate content is considered critical, as the animals leave conditions where the base of the diet was pasture, as well as all animals in finishing phase of this study, and start to receive high proportions of concentrate in the diet, generating ruminal and metabolic changes that can compromise TDMI and ADG [37].

The importance of CY in production systems in Brazil is a consequence of the marketing form used, which pays the producer according to the weight of the hot carcass and to the slaughterhouse. Therefore, animals finished in pasture, despite having presented the same CY in relation to the feedlot animals, showed lower carcass gain, which implies less economic yield [38]. This lower gain in carcass is related to the quality of the diet, since the intake of animals kept in pasture was higher than animals kept in feedlot.

The quality of the diet was directly influenced by the type of roughage (pasture or silage), as the R:C ratio between the systems was similar. Although the ME content of forage (9.40 MJ/kg DM) was higher than the corn silage (118.9 MJ/kg DM), animals finished in feedlot had higher ADG than under grazing conditions.

Considering that the ADG rate reflects the animal’s metabolic status, since it is directly associated with the amount of nutrients consumed [39], animals finished in feedlot, regardless of diet, did not show metabolic changes, a fact that contributed to the non-alteration of ADG between diets. However, animals finished in pasture showed variation in intake among the diets, but without reflecting on the ADG.

### 4.4. Effect of Finishing Systems

Due to the metabolic status of the animal, an animal prepared to metabolize a known amount of nutrients, when the intake is increased, there is a physiological addition to metabolize the largest amount of nutrients consumed. At first, it is expected that the difference and/or surplus of nutrients will support greater animal growth, and the organism tends to seek balance through a metabolic adjustment [39].

Finishing animals on pasture did not meet the high demand for nutrients from animals for carcass deposition when compared to feedlot, as verified by [40]. However, animals finished in pasture showed a higher intake NDF, which leads to a higher forage intake in the diet, that can increase the size of organs, especially the gastrointestinal tract, increasing the maintenance requirement, also due to the harvesting and grazing energy costs [14], and reducing carcass deposition [36].

Animals finished in pasture showed body weight and carcass gains of 0.99 kg/d and 0.78 kg/d, respectively. Pasture finishing animals includes the use of pasture as a fiber source, and increased carcass gain by exploring the intake of this fiber in relation to feedlot finishing [41].

Among the factors that influence the production of cattle on pasture, forage mass has the greatest impact [42]. In this study, the amount of forage mass and the forage allowance (Table 7) were determinant for the animals to perform above the observed by [43], which may be associated with the higher nutritional value of forage, especially due to its energy value.

Grazing intensity influences chemical composition of the forage [44]. The autumn/winter period and the phenotypic plasticity imposed in the period of greatest growth of the forage is decisive in modulating the pasture structure in the following period, which may imply the maturity of plant tissues, an increase in cell wall thickness and fibrous fractions [45], impairing the nutritional value.

During the finishing phase, the animal leaves puberty and there is an increase in the adipose tissue deposition and a decrease in the muscle tissue deposition [39]. These changes result in an increased energy requirement for gain, since the deposition of adipose tissue is less efficient per unit of mass than that of muscle tissue [46]. During the finishing phase, the proportion of fat deposited in the carcass is directly related to energy intake [39]. Additionally, the greater liveweight, the higher energy value of the gains, since animals present greater adiposity. These facts may imply the choice of the finishing system in feedlot or pasture, with the objective of seeking greater animal efficiency in relation to the amount of carcass gained in relation to the nutrients intake.

Although pasture system provided lower ADG in relation to feedlot system, welfare studies report that cattle kept under pastures has better results for animal, environmental and post-mortem indicators than those kept under feedlot pens, besides lower stress, health problems among animals [47] and greater preference for pastures compared to feedlot [48]. Nevertheless, our study showed that there is no difference in the potential use of DDG among pasture or feedlot.

## 5. Conclusions

The effect of replacing levels of cottonseed meal with DDG is the same for beef cattle finished in conventional feedlot or in pasture.

In the feedlot system, the beef cattle performance is better than in the grazing system. Nonetheless, there is no difference in animal performance during the adaptation period or finishing phase in both systems.

Although DDG can be an alternative to replace cottonseed meal, further studies should consider an economic analysis in feedlot or pasture system, including its market availability.

## Figures and Tables

**Table 1:** Carbohydrate and protein fractions, and ruminal degradable (RDP) and undegradable (RUP) protein of corn DDG.

Item	DDG	Cottonseed Meal
CP (g/kg DM)	289.80	390.61
EE (g/kg DM)	31.11	14.42
NDF (g/kg DM)	660.74	370.20
ADF (g/kg DM)	244.70	229.40
Carbohydrate fractions (g/kg DM)		
A+B1	115.21	453.44
B2	795.79	220.10
B3	89.00	326.46
Nitrogen fractions (g/kg CP)		
A	94.37	53.35
B1	96.73	145.00
B2	560.21	609.35
B3	71.39	107.00
C	177.20	85.30
RDP (g/kg DM)	501.00	742.73
RUP (g/kg DM)	499.00	257.27

DM: dry matter; CP: crude protein; EE: ether extract; NDF: neutral detergent fiber; ADF: acid detergent fiber; Fractions A + B1: rapid degradation soluble fraction; B2: intermediate degradation fraction; B3: very slow degradation rate; C: undegradable fraction; RDP: rumen degradable protein; RUP: rumen undegradable protein.

**Table 2:** Percentage of inclusion and chemical composition of supplements and silage in the feedlot system.

Item	Supplements			Silage
	CM	50DDG	100DDG	
Corn silage	30	30	30	-
Cottonseed Meal	18.46	9.23	-	-
Corn	48.91	47.53	46.14	-
DDG	-	9.23	18.46	-
Urea/sulfate	-	0.37	0.83	-
Mineral Supplement *	2.63	2.63	2.63	-
Kaolin	-	1.02	1.94	-
Chemical Composition (% dry matter)				
DM	71.1	71.3	71.5	29
CP	13.85	13.68	13.75	7.5
ME (MJ/kg DM)	13.75	13.77	13.72	11.89
NDF	29.57	32.61	35.65	55

DDG: dried distiller’s grains; CM: conventional supplement with corn as an energy source and cottonseed meal (CM) as a protein source; 50DDG: supplement with replacement of 50% of the CM protein source by DDG; 100DDG: supplement with 100% replacement of the CM protein source by DDG; DM: dry matter; CP: crude protein; ME: metabolizable energy; NDF: neutral detergent fiber * Warranty levels: calcium—133 g/kg; phosphorus—30 g/kg; sodium—80 g/kg; potassium—50 g/kg; magnesium—68 g/kg; sulfur—25 g/kg; copper—330 mg/kg; fluorine—500 m g/kg; manganese—950 mg/kg; zinc—1220 mg/kg; cobalt—20 mg/kg; iodine—24 m g/kg; selenium—6 g/kg; vitamin A—(min) 67,000 IU/kg; vitamin D—(min) 9500 IU/kg; vitamin E—(min) 950 IU/kg; sodium monensin—650 mg/kg.

**Table 3:** Percentage of inclusion and chemical composition of supplements and forage in the pasture finishing system.

Item	Supplements			Forage
	CM	50DDG	100DDG	
Cottonseed meal	26.37	13.18	0.00	-
Corn	69.87	67.90	65.92	-
DDG	-	13.18	26.37	-
Urea/sulfate	-	0.53	1.19	-
Mineral supplement *	3.76	3.76	3.76	
Kaolin	0.00	1.45	2.77	-
Chemical Composition (% dry matter)				
DM	88.71	89.00	89.28	38.22
CP	16.57	16.33	16.42	10.98
ME (MJ/kg DM)	14.51	14.54	14.57	9.40
NDF	18.68	23.02	27.36	69.85
EE	3.35	3.56	3.77	1.85
iNDF	5.13	4.20	3.26	35.82

DDG: dried distiller’s grains; CM: conventional supplement with corn as an energy source and cottonseed meal (CM) as a protein source; 50DDG: supplement with replacement of 50% of the CM protein source by DDG; 100DDG: supplement with 100% replacement of the CM protein source by DDG; DM: dry matter; CP: crude protein; ME: metabolizable energy; NDF: neutral detergent fiber; EE: ether extract; iNDF: indigestible neutral detergent fiber * Warranty levels: calcium—133 g/kg; phosphorus—30 g/kg; sodium—80 g/kg; potassium—50 g/kg; magnesium—68 g/kg; sulfur—25 g/kg; copper—330 mg/kg; fluorine—500 m g/kg; manganese—950 mg/kg; zinc—1220 mg/kg; cobalt—20 mg/kg; iodine—24 m g/kg; selenium—6 g/kg; vitamin A—(min) 67,000 IU/kg; vitamin D—(min) 9500 IU/kg; vitamin E—(min) 950 IU/kg; sodium monensin—650 mg/kg.

**Table 4:** Nutrients intake of cattle in pasture and feedlot finishing systems.

Item	Finishing System ^(FS)^		SEM	Diets ^(D^^)^			SEM	*p*-Value		
	Feedlot	Pasture		CM	50DDG	100DDG		FS	D	FS × D
TDMI (kg/d)	10.51 ^b^	11.05 ^a^	0.530	10.39 ^b^	11.38 ^a^	10.56 ^b^	0.790	0.0462	0.0132	0.0101
TDMI (%BW)	2.21	2.22	0.110	2.19 ^ab^	2.31 ^a^	2.14 ^b^	0.170	0.9489	0.0469	0.0591
OMI (kg/d)	9.80 ^a^	9.31 ^b^	0.480	9.10 ^b^	10.10 ^a^	9.48 ^ab^	0.720	0.0478	0.0106	0.0025
CPI (kg/d)	1.45 ^b^	1.62 ^a^	0.070	1.48 ^b^	1.61 ^a^	1.52 ^ab^	0.110	0.0004	0.0307	0.0053
NDFI (kg/d)	2.73 ^b^	4.23 ^a^	0.280	2.91 ^b^	3.84 ^a^	3.68 ^a^	0.420	<0.0001	0.0001	0.0045
iNDFI (kg/d)	0.82 ^b^	1.59 ^a^	0.120	1.14 ^b^	1.39 ^a^	1.10 ^b^	0.190	<0.0001	0.0024	0.0019
NFCI (kg/d)	5.24 ^a^	3.95 ^b^	0.170	4.91 ^a^	4.67 ^a^	4.19 ^b^	0.260	<0.0001	<0.0001	0.0746
EEI (kg/d)	0.38 ^a^	0.35 ^b^	0.010	0.34 ^b^	0.38 ^a^	0.37 ^a^	0.020	0.0027	0.0001	0.0041
MEI (MJ/d)	139.36 ^b^	160.67 ^a^	6.06	146.64 ^b^	160.86 ^a^	149.52 ^b^	8.98	0.0409	0.0252	0.0182

CM: conventional supplement with corn as an energy source and cottonseed meal (CM) as a protein source; 50DDG: supplement with replacement of 50% of the CM protein source by DDG; 100DDG: supplement with 100% replacement of the CM protein source by DDG; TDMI: total dry matter intake; OMI: organic matter intake; CPI: crude protein intake; NDFI: neutral detergent fiber intake; iNDFI: indigestible neutral detergent fiber intake; NFCI: non-fibrous carbohydrate intake; EEI: ether extract intake; MEI: metabolizable energy intake; FS: finishing system; D: diets; SEM: standard error of mean. Lowercase letters on the line differ among pasture and feedlot systems and diets when *p* < 0.05 by the Tukey test.

**Table 5:** Apparent digestibility of nutrients in cattle at pasture and feedlot finishing systems.

Item	Finishing System ^(FS)^		SEM	Diets ^(D)^			SEM	*p*-Value		
	Feedlot	Pasture		CM	50DDG	100DDG		FS	D	FS × D
TDMD (%)	68.81 ^a^	58.62 ^b^	2.77	64.11	64.23	62.42	4.15	<0.0001	0.3552	0.0682
OMD (%)	71.54 ^a^	60.80 ^b^	2.59	65.52	65.51	67.34	3.88	<0.0001	0.3848	0.2529
CPD (%)	66.48 ^a^	55.19 ^b^	3.23	61.71	59.04	61.73	4.84	<0.0001	0.272	0.2527
NDFD (%)	46.85 ^b^	52.31 ^a^	3.04	45.93 ^b^	48.22 ^b^	54.49 ^a^	4.56	0.0021	0.0008	0.7584
NFCD (%)	0.84 ^a^	78.24 ^b^	3.64	81.14	82.72	79.69	5.46	0.0041	0.3849	0.8422
EED (%)	92.37 ^a^	72.76 ^b^	1.98	82.57	81.39	83.63	2.96	<0.0001	0.2017	0.176

CM: conventional supplement with corn as an energy source and cottonseed meal (CM) as a protein source; 50 DDG: supplement with replacement of 50% of the CM protein source by DDG; 100 DDG: supplement with 100% replacement of the CM protein source by DDG; TDMD: total dry matter digestibility; OMD: organic matter digestibility; CPD: crude protein digestibility; NDFD: neutral detergent fiber digestibility; NFCD: non-fibrous carbohydrate digestibility; EED: ether extract digestibility; FS: finishing system; D: diets; SEM: standard error of mean. Lowercase letters on the line differ among pasture and feedlot systems and diets when *p* < 0.05 by the Tukey test.

**Table 6:** Beef cattle performance in two finishing systems: feedlot and pasture.

Item	Finishing System ^(FS)^		SEM	Diets ^(D)^			SEM	*p*-Value		
	Feedlot	Pasture		CM	50DDG	100DDG		FS	D	FS × D
IBW (kg)	409	407	18.39	408	409	406	27.06	0.827	0.9429	0.8651
FBW (kg)	566 ^a^	504 ^b^	24.69	533	542	529	36.33	<0.0001	0.7051	0.7622
ADG (kg/d)	1.57 ^a^	0.99 ^b^	0.100	1.26	1.33	1.25	0.150	<0.0001	0.3698	0.6205
ADGadap (kg/d)	0.98	0.82	0.218	0.93	1.04	0.73	0.320	0.1571	0.0661	0.2738
HCW (kg)	313.61 ^a^	224.63 ^b^	12.84	270.81	300.82	291.54	18.89	<0.0001	0.6089	0.6536
CY%	55.40	55.60	0.860	50.80	55.50	55.10	1.270	0.6307	0.2892	0.4305
ADGc (kg/d)	1.09 ^a^	0.78 ^b^	0.070	0.95	0.97	0.89	0.110	<0.0001	0.2665	0.9960

CM: conventional supplement with corn as an energy source and cottonseed meal (CM) as a protein source; 50DDG: supplement with replacement of 50% of the CM protein source by DDG; 100DDG: supplement with 100% replacement of the CM protein source by DDG; IBW: initial body weight; FBW: final body weight; ADG: average daily gain; ADGadap: average daily gain in adaptation period; HCW: hot carcass weight; CY: carcass yield; ADGc: daily carcass gain; FS: finishing system; D: diets; SEM: standard error of mean. Lowercase letters on the line differ among pasture and feedlot systems and diets when *p* < 0.05 by the Tukey test.

**Table 7:** Forage mass (FM), morphological fractions and forage allowance of palisade grass in pasture system.

Item	Diets			SEM
	CM	50DDG	100DDG	
FM (t DM/ha)	6.22	5.94	5.92	0.73
Forage allowance (kg DM/kg)	2.95	3.33	3.19	0.96
Green leaf (%)	8.02	9.39	6.48	3.35
Green stem (%)	13.08	15.19	11.67	4.13
Senescent leaf (%)	28.38	26.82	25.56	6.53
Senescent stem (%)	28.02	20.54	30.37	8.12

CM: conventional supplement with corn as an energy source and cottonseed meal (CM) as a protein source; 50DDG: supplement with replacement of 50% of the CM protein source by DDG; 100DDG: supplement with 100% replacement of the CM protein source by DDG; FM: forage mass; SEM: standard error of mean.

## Data Availability

Data available on request due to restrictions eg privacy or ethical.
